# Another College of Dental Surgery Contemplated

**Published:** 1852-01

**Authors:** 


*Another College of Dental Surgery Contemplated.*—In a series of spiritedly written articles, published in a Nashville paper, the peculiar advantages of that location for a school of dentistry, are ably urged by Dr. B. Wood, surgeon dentist. Although it would give us pleasure to see a college of dental surgery in successful operation in Tennessee, we fear the undertaking would not, at present, meet with sufficient encouragement to justify men, properly qualified as teachers, in devoting the necessary amount of time and labor to the subject. We admire the enthusiasm and ardent desire for the elevation of the profession, manifested by the writer of these articles, and most sincerely do we wish the attainment of the object contemplated by them, were possible, but with our knowledge of the profession in the United States, together with the experience we have had in teaching, we believe it would require fifteen years of unremitting labor to secure a class of fifteen students. We do not say this to discourage the praiseworthy and commendable efforts of Dr. Wood, but because we fear that, if a school of dentistry were established in Nashville, at this time, sufficiently large classes could not be drawn together to justify the continuance of the labors of those who might become associated in the enterprize. Until the colleges of dental surgery now in existence in the United States are better sustained, we are inclined to doubt the propriety, and we are not influenced in the expression of this opinion, by any unkind or selfish motive, of establishing others.

Collegiate instruction in dental surgery is a new thing, and although the experiment has been sufficiently tested to convince those who have carefully examined the matter, that it will ultimately become indispensable, yet it will be some time before that period arrives. When it does come, the enterprise of American dentists, will not long permit the want to remain unsupplied.

